# Influence of printing orientation and post‐curing on optical and surface properties of definitive three‐dimensional‐printed dental resin with inorganic fillers

**DOI:** 10.1111/eos.70083

**Published:** 2026-03-09

**Authors:** Luana dos Santos Souza, Andrea Mendoza, Taciana Marco Ferraz Caneppele, Pablo Lenin Benitez Sellan, Eduardo Bresciani

**Affiliations:** ^1^ Department of Restorative Dentistry Institute of Science and Technology São Paulo State University (UNESP) São José dos Campos Brazil; ^2^ Department of Restorative Dentistry and Prosthodontics School of Dentistry Universidad Espíritu Santo Samborondón Ecuador

**Keywords:** additive manufacturing, computer‐aided design, dental crown, dental materials

## Abstract

This study evaluated the influence of printing orientation and post‐curing on the color stability, gloss retention, and surface microhardness of a definitive three‐dimensional (3D)‐printed dental resin, a high‐strength photopolymer with inorganic fillers designed for permanent restorations, after immersion in a staining solution. Circular specimens (6 mm diameter and 1.5 mm thickness) were printed at 0°, 45°, and 90° orientations and divided into two groups: control (*n* = 10), post‐cured according to the manufacturer's protocol, and negative control (*n* = 10), without post‐curing. Baseline color (*L* ^*^, *a* ^*^, *b* ^*^), gloss, and Knoop microhardness were measured, followed by reassessment after 21 days of immersion in a staining solution. Data were analyzed using two‐way and repeated‐measures analysis of variance (*α* = 0.05). Non‐post‐cured specimens showed perceptible but clinically acceptable color changes. Microhardness decreased after staining in all groups, regardless of curing. Gloss significantly diminished post‐staining, with the control group printed at 45° showing superior retention. Printing orientation influenced color stability, with the 0° orientation showing the highest stability. Post‐curing enhanced color stability and gloss retention in definitive 3D‐printed resins. Printing orientation affected optical outcomes, emphasizing the importance of optimizing both parameters for long‐term esthetic and mechanical reliability.

## INTRODUCTION

Three‐dimensional (3D) printing has emerged as an innovative technology in dentistry, offering enhanced precision, customization, and efficiency across various clinical and laboratory procedures [1]. Since its inception in the 1980s, leveraging the photopolymerization of polymeric resins, 3D printing has undergone significant advancements, enabling the fabrication of intricate and patient‐specific structures with remarkable accuracy and reproducibility [2]. This innovative approach has been increasingly adopted in restorative dentistry for the creation of permanent crowns, driven by its advantages in terms of accuracy, cost‐effectiveness, and design versatility [3].

Dental crowns, as prosthetic restorations covering the visible portion of a tooth, play a critical role in restoring function, strength, and aesthetics [3]. The long‐term success of these restorations hinges on their ability to withstand the oral environment, including masticatory forces, while maintaining a satisfactory appearance [4]. Among the key aesthetic parameters influencing patient and clinician acceptance are color stability and surface gloss [5]. Understanding how these properties are affected by factors encountered in daily use is paramount for the development of more durable and aesthetically reliable dental materials [6].

Despite the rapid advancements in 3D printing for dental applications, the final quality of printed restorations can still be influenced by various technical parameters, including printer type, material selection, printing orientation, and post‐processing procedures such as additional curing [7]. A significant challenge associated with 3D‐printed resins for permanent crowns is their potential for discoloration and degradation of aesthetic properties over time [8]. Exposure to common staining agents found in the oral environment, such as coffee, tea, red wine, and certain foods and beverages, can negatively impact the color stability and gloss of these resins [9]. This phenomenon arises from the interaction of chromogens within these solutions with the resin surface, leading to color alterations and a reduction in surface luster, ultimately compromising the clinical efficacy and patient acceptance of the restorations [10, 11].

Furthermore, the printing direction, or the orientation of the restoration on the build platform, has been shown to significantly affect the dimensional accuracy, mechanical strength, and surface quality of printed restorations [12]. Despite the rapid progress of additive manufacturing in dentistry, there is still limited evidence on how printing orientation and post‐curing protocols affect the optical performance of resin‐based materials. Most published studies have emphasized mechanical properties, such as flexural strength and hardness, whereas investigations addressing optical parameters like color stability, gloss, and translucency remain comparatively scarce [12]. Recent findings indicate that printing orientation can significantly influence translucency and color in 3D‐printed restorative resins [13], and that both orientation and post‐curing time can alter color stability and translucency in temporary restorations [14]. Furthermore, very few studies have examined the optical behavior of resins formulated with a high content of inorganic fillers, even though these fillers may substantially affect esthetic outcomes by modifying light scattering and surface interactions. This gap highlights the relevance of investigating the combined effects of printing orientation and post‐curing on the optical properties of resin materials intended for definitive dental restorations.

Therefore, this study aims to evaluate the combined effect of printing orientation and post‐curing on the color stability, gloss retention, and surface microhardness of 3D‐printed dental resins with a high content of inorganic fillers, designed for definitive restorations. Two null hypotheses were tested: (1) printing orientation does not significantly influence these optical and surface properties; and (2) the absence of post‐curing does not significantly influence these optical and surface properties.

## MATERIAL AND METHODS

Based on the findings of de Castro et al. [15] and using the mean gloss values previously reported for 3D‐printed resin (ranging from 28.9 ± 10.9 gloss unit [GU]), a power analysis was performed using g*power software (Heinrich‐Heine‐Universität Düsseldorf). Considering a significance level (*α*) of 5% and a power (1 − *β*) of 80%, the calculated minimum sample size was 10 specimens per group. With this, a total of 60 disc‐shaped specimens (6.0 mm in diameter × 1.5 mm in thickness) were fabricated to evaluate the effect of printing orientation and post‐curing protocol on the color stability, gloss retention, and surface microhardness of a 3D‐printed dental resin.

**FIGURE 1:**
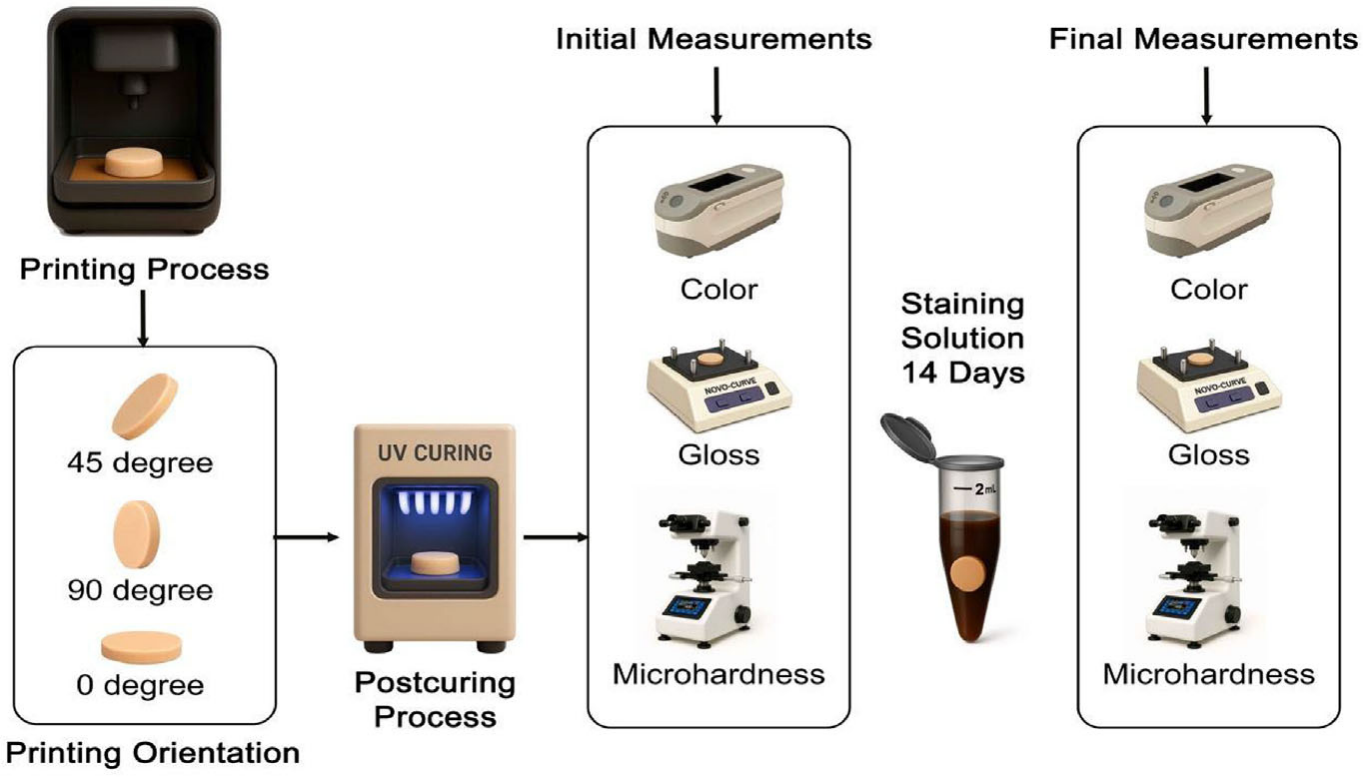
Schematic diagram illustrating the methodology and group allocation. UV, ultraviolet.

The specimens were printed at three building orientations (0°, 45°, and 90°) and divided into two post‐curing conditions: (1) specimens subjected to the manufacturer's recommended cure using a preprogrammed material profile (*n* = 10) and (2) specimens undergoing no post‐cure beyond the initial standard protocol (*n* = 10). A schematic graphical abstract illustrating the specimen fabrication, post‐processing, and analytical procedures is presented in Figure 1.

The discs were designed using cad software (Rhinoceros; Robert McNeel & Associates) and exported in STL format. Slicing was performed in the proprietary software (RayWare; SprintRay) with a fixed layer thickness of 100 µm. Printing was carried out using a digital light processing 3D printer (SprintRay Pro 95S) and a permanent crown resin composed of methacrylate monomers and oligomers, acrylic monomers, photoinitiators, and inorganic fillers, with the total content of inorganic fillers exceeding 50% by mass (Ceramic Crown; SprintRay). After printing, all specimens were cleaned in 90% isopropyl alcohol using an automated unit (Pro Wash/Dry; SprintRay) for 5 min. The specimens were then divided into two groups: the control group, which underwent the manufacturer's recommended post‐curing cycle in an ultraviolet (UV) curing unit for 9 min (ProCure 2; SprintRay) (*n* = 10), and the negative control group, which did not receive any post‐curing protocol beyond the initial cleaning step (*n* = 10). The inclusion of the negative control group aimed to provide a baseline for comparison and to demonstrate the importance of the manufacturer's post‐curing protocol. After these procedures, the support structures of all specimens were removed using cutting pliers.

All specimens (*n* = 10) were polished on both sides using a polishing machine (Polipan‐U; Panambra) at 600 rpm with silicon carbide abrasive papers (Silicon Carbide; Extec) in a decreasing grit sequence: 15 µm (P1200—30 s), 10 µm (P2400—60 s), and 5 µm (P4000—120 s), under copious water cooling. Between abrasive paper changes and at the end of the polishing procedure, the specimens were cleaned in an ultrasonic bath for 10 min and were standardized and polished using a manual polishing machine with a sequential series of abrasive papers. Following this preparation, baseline measurements of color, gloss, and microhardness were performed.

For color assessment, the original color of each sample was evaluated under controlled environmental conditions, according to the CIE Lab (Commission Internationale de l'Eclairage) system, using a spectrophotometer (CM 2600d; Konica Minolta) with an integrating sphere. The device was set to use the standard illuminant D65 with 100% UV light and the specular component included. The observation angle was set at 10°, and the device was adjusted to a small aperture viewing. The color of each sample was measured three times, and the results were averaged. The color measurement results were quantified concerning three coordinate values (*L* ^*^, *a* ^*^, *b* ^*^), as established by the CIE, which locates the color of an object in a 3D color space.

To standardize the specimen position and ensure the light beam consistently reached the same location, white supports measuring 7.0 mm × 7.0 mm and 2.0 mm in depth were utilized. A demarcation was made on the posterior portion of each sample to standardize its insertion into the color analysis device. The color difference (Δ*E*_00_) was calculated using the CIEDE2000 formula. The perceptibility threshold (PT) of 0.8 and the acceptability threshold (AT) of 1.8 were adopted in this study [16].

Gloss measurements were performed using the Novo‐Curve device, which operates with a reading area of 2 mm ×2 mm and a 60° geometry for light incidence, presenting results in GUs. To minimize ambient light interference, a metal positioner that blocked ambient light was employed. Each specimen was measured three times randomly, and the average of these measurements was taken as the spectral gloss value.

The Knoop microhardness of the specimens was evaluated using a microhardness tester (FM‐700; Future‐Tech) programmed with a load of 0.245 N for 10 s, with three indentations made on each specimen to determine the mean and standard deviation. After the initial measurements, the specimens were immersed in Eppendorf tubes containing 2 mL of staining solutions (prepared by dissolving 1.5 g of finely ground instant coffee, 1.5 g of finely ground instant black tea, 0.333 mL of FD&C Yellow 5, 0.125 g of methylparaben, 0.075 g of propylparaben, and 41.6 mL of red wine in 0.5 L of distilled water) and subjected to constant agitation for 21 days [17]. Following the immersion period, the specimens were washed in distilled water for 60 s, and the color was assessed. Subsequently, the final measurements of gloss, microhardness, and color were analyzed.

The results were tested for homogeneity with the Levene test and for normality with the Shapiro–Wilk test. A 2‐way analysis of variance (ANOVA) was used for Δ*E*_00_ (print direction and curing process). A 3‐way repeated measures ANOVA was used for the microhardness and gloss (print direction, curing process, and time). ANOVA tests were followed by the Tukey's test (*α* = 0.05).

## RESULTS

The analysis of color differences (Δ*E*_00_) revealed statistically significant effects for both printing orientation (*p* = 0.011) and curing condition (*p* < 0.001), as well as for their interaction. Regarding this interaction, specimens printed at 45° showed no significant color change differences between the control (1.396 ± 0.273) and negative control (1.573 ± 0.253) groups (*p* = 0.977). In contrast, the curing process significantly influenced color stability in specimens printed at 90° (control: 1.129 ± 0.182; negative control: 1.529 ± 0.188; *p* < 0.001) and 0° (control: 0.870 ± 0.242; negative control: 1.636 ± 0.278; *p* < 0.001), with the negative control groups consistently exhibiting higher Δ*E*_00_ values. Notably, color changes in the 90° control group were comparable to those observed in negative control specimens printed at 45° and 0°. Within the control group, printing orientation did not produce statistically significant differences in color alteration (*p* = 0.699).

Despite these statistical differences, all groups remained below the clinical AT (Δ*E*_00_ = 1.8). Moreover, the mean color change for the 0° control group (0.870 ± 0.242) was below the PT (Δ*E*_00_ = 0.8), indicating visually imperceptible color differences under standard conditions.

GU values demonstrated statistically significant differences in the triple interaction between time, printing direction, and curing process (*p* < 0.001). Specifically, specimens in the negative control group had lower gloss compared to specimens in the control group (*p* < 0.001). Additionally, a generalized decrease in spectral gloss was observed across all groups after 21 days of staining solution exposure, irrespective of printing direction or curing process (*p* < 0.001). In specimens in the no post‐cure group, the printing direction influenced the gloss, with specimens printed at 45° showing higher final gloss (Table 1; *p* = 0.003). Finally, analysis of the final gloss revealed that specimens printed at 45° were not affected by the curing process, demonstrating similar behavior between the no post‐cure group and the manufacturer recommended cure group (*p* = 0.999).

**TABLE 1:** Mean and standard deviation of the interaction between time, printing direction, and curing process.

	Control group	Negative control group
**Initial gloss**		
45°	85.2 ± 2.62^Aa^	78.6 ± 4.94^Aa^ ^*^
90°	85.6 ± 1.89^Aa^	74.8 ± 2.97^ABa^ ^*^
0°	82.3 ± 1.94^Aa^	73.3 ± 3.39^Ba^ ^*^
**Final gloss**		
45°	47.5 ± 8.20^Ab^	52.2 ± 9.57^Ab^
90°	56.5 ± 2.73^Ab^	35.0 ± 2.13^Bb^ ^*^
0°	49.0 ± 8.34^Ab^	28.2 ± 5.96^Bb^ ^*^

*Note*: Different letters indicate statistically significant differences. Uppercase letters—comparisons between printing angles at the same time point and with the same curing process. Lowercase letters—comparisons between time points at the same printing direction and with the same curing process.

^*^ Asterisks denotes a statistically significant difference in the comparison between curing processes at the same time point and printing angle.

Regarding the variations in the GU, the specimens in the control group and printed at 45° presented a ΔGU of 38.2. At printing angles of 90° and 0°, the ΔGU values were 29.1 and 33.3, respectively. In contrast, the specimens in the negative control group presented ΔGU values of 26.4 (45°), 39.8 (90°), and 45.1 (0°).

Microhardness analysis revealed a statistically significant difference in the temporal factor, with a decrease observed at the final time point. Furthermore, a significant interaction was found between the temporal factors (initial and final) and the curing process. Specifically, the final microhardness was significantly influenced by the curing process (*p* < 0.001), samples in the negative control group showing lower values compared to control group samples (Table 2). The printing direction had no influence on the microhardness.

**TABLE 2:** Mean and standard deviation values of the interaction between time and the curing process.

	Control group	Negative control group
Initial KNH	52.1 ± 3.35^Aa^	53.1 ± 4.46^Aa^
Final KNH	37.0 ± 3.03^Ba^	28.8 ± 4.73^Bb^

*Note*: Distinct letters indicate statistical differences, with uppercase letters denoting comparisons between time and lowercase letters denoting comparisons between curing process.

The general linear models (GLMs) confirmed that curing condition was the main factor influencing all evaluated properties (*p* < 0.001). Printing orientation also showed a significant effect on color stability (*p* = 0.011), gloss retention (*p* < 0.001), and microhardness (*p* < 0.001). Significant interaction effects between orientation and curing condition were observed for Δ*E*_00_, gloss, and microhardness, indicating that the influence of printing orientation was dependent on whether specimens were subjected to UV post‐curing. Detailed results of the GLMs are provided in Tables S1–S3.

## DISCUSSION

The first and second null hypothesis cannot be accepted. Among the evaluated parameters, only microhardness was not significantly influenced by the printing direction.

3D printing enables the layer‐by‐layer fabrication of resin‐based components and is recognized for its efficiency and material‐saving benefits [18]. Despite manufacturers classifying 3D‐printed resins as definitive materials, comprehensive evaluation of their properties, especially those related to clinical performance and longevity, is essential. Key influencing factors include printing orientation, layer thickness, and degree of polymerization [19, 20].

The tested parameters (color stability, microhardness, and gloss) are considered relevant indicators of long‐term success in restorative treatments. According to ISO/TR 28,642:2016, color changes should be evaluated under 50%:50% visual perception conditions. In this study, color stability was assessed using the CIEDE2000 metric, which is more clinically sensitive and reliable for detecting perceptible changes [16].

Microhardness, gloss, and color variation have been suggested as indirect markers of material degradation, influencing the survival rate of dental restorations [21]. In this study, the group without post‐curing was intentionally included as a negative control to demonstrate the importance of the manufacturer's recommended curing protocol. As expected, these specimens consistently showed inferior performance, with more pronounced color change and gloss loss after staining. These findings are in line with those of Lee et al. [22], who reported that uncured materials exhibit increased dye uptake and chromatic instability, likely due to unreacted monomers and incomplete polymerization. The inferior results of the negative control group in the present study reinforce that omitting the post‐curing cycle is not a clinically acceptable condition but serves as a baseline to highlight the benefits of proper curing.

Additionally, Espinar et al. [13] reported that printing orientation significantly affects optical characteristics, particularly lightness and chroma. In this study, although all groups showed perceptible discoloration after 21 days of staining, the changes remained within acceptable limits. The magnitude of these changes likely reflects the staining protocol and type of solution used. Notably, cured specimens consistently exhibited better color stability, supporting the notion that post‐curing enhances the polymer matrix and reduces susceptibility to discoloration over time [22].

The decrease in microhardness after staining may be attributed to water and dye sorption, leading to matrix plasticization and disruption of polymer crosslinking. This compromises structural integrity and increases vulnerability to wear and chemical degradation. Although this mechanism has also been reported in conventional composite resins, the effects tend to be less severe due to the presence of inorganic fillers that reduce water sorption and enhance mechanical properties [23].

Furthermore, although lower radiant exposure is known to reduce microhardness by limiting the conversion of carbon–carbon double bonds, the final microhardness results found in this study favor the findings already reported in the literature, demonstrating the influence of the curing process on surface microhardness [24].

**FIGURE 2:**
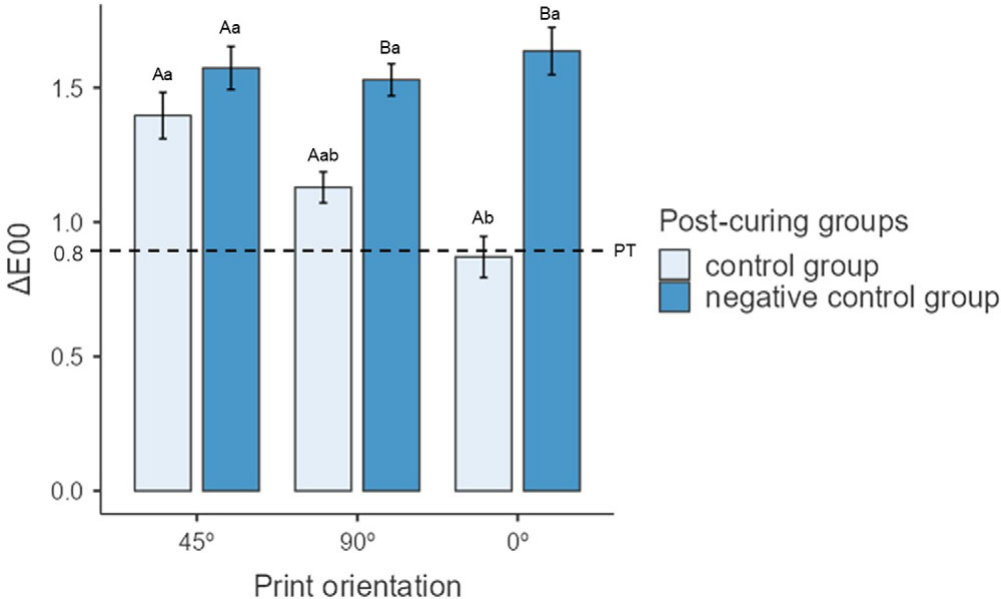
Analysis of color change (Δ*E*_00_) by print orientation and post‐curing condition after coloring. Distinct letters indicate statistical differences, with uppercase letters denoting comparisons between curing processes and lowercase letters denoting comparisons between printing directions.

Regarding gloss, most groups exceeded the clinical AT (ΔGU = 34.2) under D65 illuminant, except for cured specimens printed at 90° and uncured specimens printed at 45°, which remained within acceptable limits. According to Rocha et al. [25], gloss changes above ΔGU = 7.0 are perceptible, and the results highlight the influence of both curing condition and print angle on surface appearance. These findings contrast with those of de Castro et al. [15], who reported no effect of print orientation on gloss, roughness, or color. Such discrepancies may arise from methodological differences, this study included a staining protocol that may have amplified the visual and surface changes (Figure 2).

Several authors have emphasized the importance of printing orientation due to the anisotropic behavior of 3D printing, which affects the physical properties of the final object [26, 27]. Yang et al. [28] and others recommend 45° orientation for improved marginal fit, with reports of acceptable intraoral performance at this angle. Conversely, de Castro et al. [29] argue that printing at 90 angle may improve accuracy by reducing the “staircase effect” introduced during slicing at 45 angle.

However, our results suggest that post‐curing had a greater influence on material properties than the printing direction. Specimens in the manufacturer recommended cure group consistently outperformed specimens in the no post‐cure group in most parameters. Although the 45° orientation is often preferred in literature, in our study, this printing direction showed lower color stability, still within acceptable limits. Gloss was more affected by the printing angle in the no post‐cure group, where specimens printed at 45° maintained better gloss, as expected.

As Bataweel et al. [30] pointed out, the composition of 3D‐printed resins still requires optimization to ensure the durability of aesthetic parameters such as color and gloss stability. The decrease in microhardness in this study demonstrates the surface degradation observed after staining and reinforces the need to optimize post‐curing protocols.

The evaluation of the properties of 3D‐printed resins reveals a complex interaction between printing orientation and post‐curing, with post‐curing demonstrating a more consistent impact on improving color stability and gloss, whereas microhardness was less sensitive to printing direction. Discoloration after staining, although within clinical limits (Δ*E*_00_ < 1.8), was more pronounced in specimens in the no post‐cure group, highlighting the importance of additional curing for the integrity of the polymer matrix. The decrease in microhardness after staining, possibly due to water and dye sorption, signals a potential vulnerability to long‐term degradation. The discrepancies observed regarding the influence of printing orientation in the literature suggest the need for future studies that standardize protocols and simulate intraoral conditions for a clearer understanding and optimized clinical workflows with 3D printed dental restorations.

The GLMs confirmed that the curing condition was the factor with the greatest impact on all outcomes, whereas printing orientation had a secondary but significant influence on color stability and gloss. This finding is consistent with Espinar et al. [13], who reported that orientation mainly affects chroma and translucency, but less so mechanical performance, and with de Castro et al. [15], who found variable effects of build orientation on surface gloss and color depending on experimental design. The significant interaction between curing condition and orientation observed in the present study highlights that the effect of orientation cannot be interpreted in isolation, as it depends on whether the material was adequately post‐cured. These results underscore the novelty of investigating both variables simultaneously in definitive resins with high inorganic filler content, a field still scarcely explored.

The findings of this study highlight clinically relevant considerations for the use of 3D‐printed dental restorations. The superior color stability observed in specimens printed at a 0° orientation suggests that this angle should be prioritized in cases where esthetic outcomes are critical. Surface gloss was better preserved in specimens printed at a 45° angle with post‐curing, which may be relevant in scenarios where a post‐curing protocol cannot be performed. Microhardness was not significantly affected by the printing angle, indicating that mechanical strength is likely to remain consistent regardless of build orientation. Overall, the curing protocol had a more pronounced influence on the final properties of the printed materials than the printing direction, emphasizing the need for standardized post‐curing procedures to ensure the clinical reliability and long‐term performance of 3D‐printed restorations.

## AUTHOR CONTRIBUTIONS

**Conceptualization**: Luana dos Santos Souza, Andrea Mendoza, Taciana Marco Ferraz Caneppele, Pablo Lenin Benitez Sellan, and Eduardo Bresciani. **Methodology**: Luana dos Santos Souza, Andrea Mendoza, Taciana Marco Ferraz Caneppele, Pablo Lenin Benitez Sellan, and Eduardo Bresciani. **Formal analysis**: Luana dos Santos Souza, Andrea Mendoza, Taciana Marco Ferraz Caneppele, Pablo Lenin Benitez Sellan, and Eduardo Bresciani. **Data curation**: Luana dos Santos Souza, Taciana Marco Ferraz Caneppele, Pablo Lenin Benitez Sellan, and Eduardo Bresciani. **Writing—original draft**: Luana dos Santos Souza, Taciana Marco Ferraz Caneppele, Pablo Lenin Benitez Sellan, and Eduardo Bresciani. **Writing—review and editing**: Luana dos Santos Souza, Taciana Marco Ferraz Caneppele, Pablo Lenin Benitez Sellan, and Eduardo Bresciani. **Visualization**: Luana dos Santos Souza, Taciana Marco Ferraz Caneppele, Pablo Lenin Benitez Sellan, and Eduardo Bresciani. **Investigation**: Andrea Mendoza. **Project administration**: Pablo Lenin Benitez Sellan and Eduardo Bresciani. **Resources**: Pablo Lenin Benitez Sellan and Eduardo Bresciani. **Supervision**: Eduardo Bresciani.

## CONFLICT OF INTEREST STATEMENT

The authors declare no conflicts of interest.

## Supporting information



Supporting Information
