# Supplementary material for: Influence of printing orientation and post‐curing on optical and surface properties of definitive three‐dimensional‐printed dental resin with inorganic fillers
Source: Eur J Oral Sci. 2026 Mar 9;134(4):e70083. doi: 10.1111/eos.70083 (PMC13377923; doi:10.1111/eos.70083)
Supplement: Supplementary file 1 — Supporting Information [file EOS-134-e70083-s001.docx]

**SUPPLEMENTARY MATERIAL**

Table 1. GLM for color stability (ΔE00).

| **Predictor** | **F-value** | **p-value** |
| --- | --- | --- |
| Print orientation | 4.87 | 0.0011* |
| Curing condition (Control vs Negative control) | 52.62 | <0.001*** |
| Orientation × Curing condition | 7.74 | 0.001** |

Table 2. GLM for gloss retention (GU)

Intra-subject effects (time-related factors):

| **Predictor** | **F-value** | **p-value** |
| --- | --- | --- |
| Time | 1324.00 | <0.001*** |
| Time x Orientation | 4.75 | 0.013 * |
| Time x Curing condition | 3.74 | 0.058 |
| Time x Orientation x Curing condition | 15.13 | <0.001 *** |

Inter-subject effects (between groups):

| **Predictor** | **F-value** | **p-value** |
| --- | --- | --- |
| Print orientation | 21.9 | <0.001 *** |
| Curing condition (control x negative control group) | 122.7 | <0.001 *** |
| Orientation x Curing condition | 25.8 | <0.001 *** |

Table 3. GLM for microhardness (KNH)

Intra-subject effects (time-related factors):

| **Predictor** | **F-value** | **p-value** |
| --- | --- | --- |
| Time | 751.05 | <0.001 *** |
| Time x Orientation | 3.09 | 0.054 |
| Time x Curing condition | 41.28 | <0.001 *** |
| Time x Orientation x Curing condition | 0.43 | 0.651 |

Inter-subject effects (between groups):

| **Predictor** | **F-value** | **p-value** |
| --- | --- | --- |
| Print orientation | 14.6 | <0.001 *** |
| Curing condition (control x negative control group) | 53.7 | <0.001 *** |
| Orientation x Curing condition | 19.8 | <0.001 *** |
